# Biodegradation of atrazine and ligninolytic enzyme production by basidiomycete strains

**DOI:** 10.1186/s12866-020-01950-0

**Published:** 2020-08-26

**Authors:** Caroline Henn, Diego Alves Monteiro, Mauricio Boscolo, Roberto da Silva, Eleni Gomes

**Affiliations:** 1 ITAIPU Binacional, Divisão de Reservatório, Avenida Tancredo Neves, 6731, Foz do Iguaçu, PR 85866-900 Brasil; 2 Laboratório de Bioquímica e Microbiologia Aplicada, Instituto de Biociências, Letras e Ciências Exatas, Universidade Estadual Paulista, UNESP, São José do Rio Preto, SP Brasil

**Keywords:** Fungal metabolism, Biodegradation, Laccase, Organochlorinated, Rainforest fungi, Basidiomycete, Co-metabolism

## Abstract

**Background:**

Atrazine is one of the most widespread chlorinated herbicides, leaving large bulks in soils and groundwater. The biodegradation of atrazine by bacteria is well described, but many aspects of the fungal metabolism of this compound remain unclear. Thus, we investigated the toxicity and degradation of atrazine by 13 rainforest basidiomycete strains.

**Results:**

In liquid medium, *Pluteus cubensis* SXS320, *Gloelophyllum striatum* MCA7, and *Agaricales* MCA17 removed 30, 37, and 38%, respectively, of initial 25 mg L^− 1^ of the herbicide within 20 days. Deficiency of nitrogen drove atrazine degradation by *Pluteus cubensis* SXS320; this strain removed 30% of atrazine within 20 days in a culture medium with 2.5 mM of N, raising three metabolites; in a medium with 25 mM of N, only 21% of initial atrazine were removed after 40 days, and two metabolites appeared in culture extracts. This is the first report of such different outcomes linked to nitrogen availability during the biodegradation of atrazine by basidiomycetes. The herbicide also induced synthesis and secretion of extracellular laccases by *Datronia caperata* MCA5, *Pycnoporus sanguineus* MCA16, and *Polyporus tenuiculus* MCA11. Laccase levels produced by of *P. tenuiculus* MCA11 were 13.3-fold superior in the contaminated medium than in control; the possible role of this enzyme on atrazine biodegradation was evaluated, considering the strong induction and the removal of 13.9% of the herbicide in vivo. Although 88% of initial laccase activity remained after 6 h, no evidence of in vitro degradation was observed, even though ABTS was present as mediator.

**Conclusions:**

This study revealed a high potential for atrazine biodegradation among tropical basidiomycete strains. Further investigations, focusing on less explored ligninolytic enzymes and cell-bound mechanisms, could enlighten key aspects of the atrazine fungal metabolism and the role of the nitrogen in the process.

## Background

Agricultural production is strongly dependent on agrochemicals. Atrazine (2-chloro-4-ethylamino-6-isopropylamino)-1,3,5-triazine) is the second most employed herbicide worldwide, used to control broadleaf weeds in sugarcane and maize plantations. It has considerable persistence, with a half-life of around 41–231 days, and high solubility and mobility in soils, raising concerns about groundwater contamination. As a result, atrazine may enter the food chain, affecting biodiversity and human health [1–3]. Atrazine belongs to the chemical class of chlorinated triazines, and is formed by a heterocyclic aromatic ring alternating carbon and nitrogen atoms, besides chlorine substituent. Like other halogenated aromatic compounds, atrazine and its dealkylated and deaminated metabolites are toxic, carcinogenic and endocrine disruptors [3, 4].

Once lignin polyphenolic structure presents similarities with many aromatic pollutants, microbial ligninolytic systems have been extensively studied in the last years, searching for potential tools for bioremediation of contaminated sites and for a better understanding of the environmental fate of toxic compounds [5, 6]. Recent proteomic approaches concluded that wood decay, itself, is a source of toxic phenolic derivatives and reactive oxygen species; thus, ligninolytic and stress response enzymes are expressed simultaneously [7]. White-rot fungi are the main producers of non-specific oxidases that can act upon a wide range of substrates. Laccases (Lac EC 1.10.3.2), lignin peroxidases (LiP; EC 1.11.1.14) and manganese peroxidases (MnP; EC 1.11.1.13) are responsible for the degradation of the most recalcitrant molecule in nature [8]. Thus, the co-metabolic biodegradation of pesticides, PAHs (polycyclic aromatic hydrocarbons), polychlorinated phenols, polymers, dioxins, pesticides, and dyes by ligninases have been subject of intense research [9–13].

A complete catabolic pathway for atrazine biodegradation was already described for bacteria (e.g., *Pseudomonas* strain ADP). The plasmidial genes atzA, atzB, and atzC encode hydrolytic enzymes, responsible for atrazine mineralization [14]. Conversely, fungal biodegradation is based on oxidative-hydrolytic mechanisms and until now, the metabolic routes identified are more diverse. Fungi usually modify the molecule by sequentially removing aromatic ring substituents, with dealkylation as first step [15, 16]. The resulting metabolites, such as deethylatrazine (DEA), deisopropylatrazine (DIA), deethyldeisopropylatrazine (DEA-DIA), and hydroxyatrazine (HA) still retain the aromatic ring [16, 17]. The degradation of atrazine derivatives DIA and DEA by a fungal strain (*Pleurotus ostreatus* INCQS 40310) was only recently described [4].

Despite the limitations imposed by the incomplete atrazine biodegradation by basidiomycetes in pure cultures, studies applying purified ligninolytic enzymes under controlled conditions, or adopting co-cultures involving soil bacteria and deuteromycetes, have been successful in achieving complete dissipation of atrazine and detoxification of contaminated substrates [4, 18, 19]. White-rot fungi bioremediation approaches also present the advantage of being low cost processes, once fungal species are usually not fastidious; being mainly saprophytes, their growth can be easily supported by the organic residues, in laboratory culture conditions or in the field [20]. Among the few nutritional exigencies to observe, nitrogen availability plays a central role; when ligninolytic enzymes are responsible for the biodegradation, it may be necessary to establish nitrogen-starving conditions. On the other side, cell bound mechanisms are favored by nutrient rich medium, supporting an extensive biomass development. In this sense, a previous exploration of metabolic profile of indigenous fungal strains is critical during the prospection of species for this purpose [21–23].

The current knowledge about fungal biodegradation of xenobiotics and the role of ligninolytic enzymes is based on the findings for a few well studied basidiomycete strains, mainly *Pleurotus pulmonarius*, *Phanerochaete chrysosporium*, *Lentinus* spp., *Pleurotus* spp. and *Trametes* spp. [5, 16, 20, 21]. Some of the most recent estimates about fungal biodiversity suggest the existence of 2.2 to 3.8 million species, being only 120,000 currently described [24]. Therefore, a wide frontier in microbial diversity - and its biotechnological potential - remains unexplored. Tropical rainforests in South America are considered biodiversity hotspots, and account for much of this gap. In this work, we screened fungal strains, isolated from tropical rainforest fragments, for their capacity of growing in the presence of atrazine and metabolizing it. We also observed the profile of secondary metabolites arising from atrazine degradation under different culture conditions, while searching for a possible role of ligninolytic enzymes and nitrogen availability in the process.

## Results

**Fig. 1:**
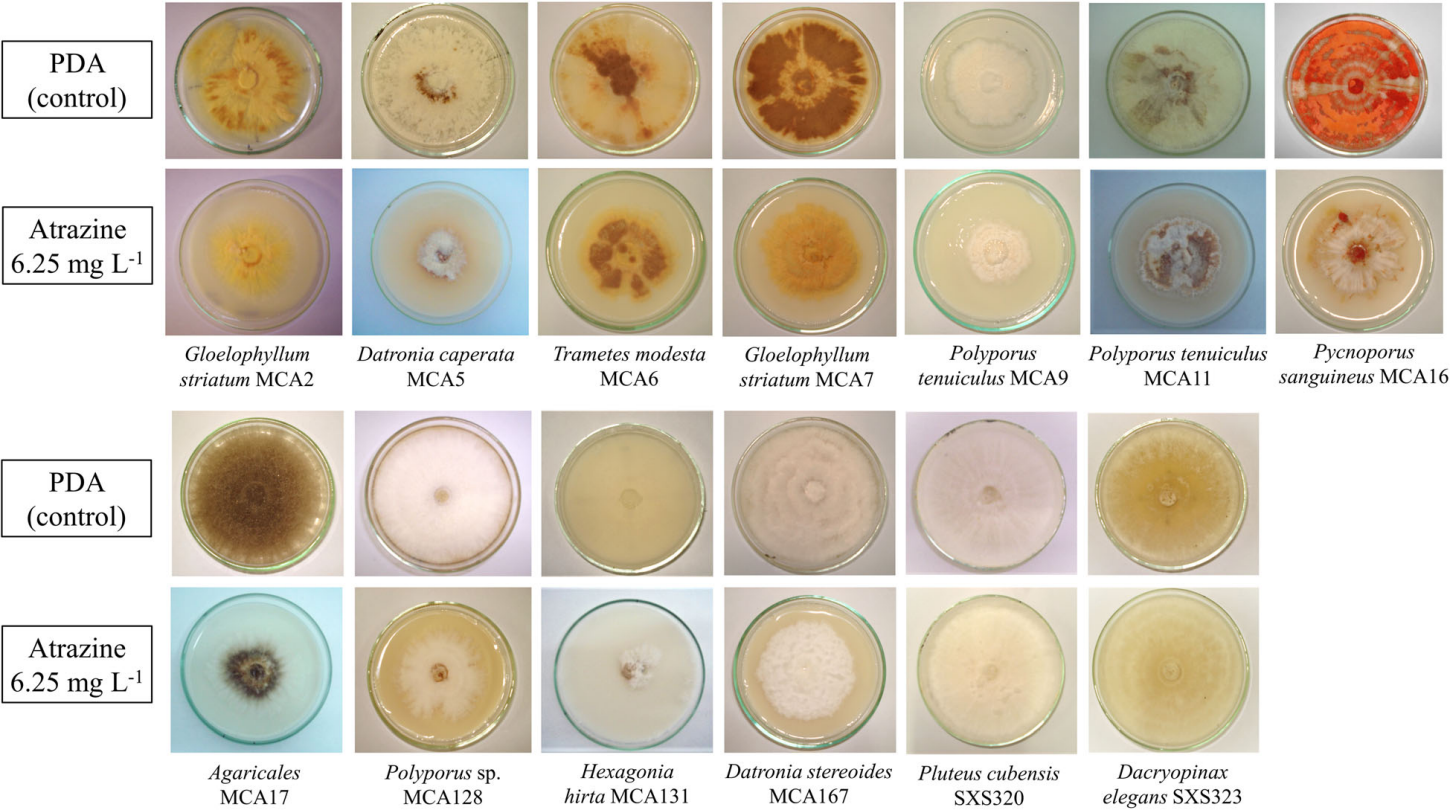
Fungal strains after 15 days of cultivation in PDA medium. Two plates are shown for each strain: control (above) and the medium containing 6.25 g L^− 1^ of atrazine (below). The cultures containing 10 g L^− 1^ of atrazine, very similar to the last ones, were omitted

Although all microorganisms studied here were sensitive to atrazine to some degree, all could grow. Inhibition rates were very similar for the two atrazine concentrations (6.25 g L^− 1^ and 10 g L^− 1^, Table 1), and always distinct from the control; except for *P. tenuiculus* MCA11, for which a difference (*p* ≤ 0.05) was observed between distinct atrazine levels. At 6.25 g L^− 1^, we found minor inhibitory effect for *Polyporus tenuiculus* MCA9 (42%) and *Pluteus cubensis* SXS320 (44%); conversely, the most affected strains were *Polyporus* sp. MCA128 and *Datronia stereoides* MCA167, which showed inhibition of 72 and 73%, respectively. The fungal mycelium of some strains was much denser in the contaminated medium when compared to control; this was the case of *Gloellophylum striatum* MCA7. Many strains also differed in pigmentation (Fig. 1).

**Table 1:** Growth inhibition of fungal strains in PDA medium containing atrazine. M.D.: mycelium density; weak (1); medium (2); high (3). Superscript lowercase letters (a, b, and c) refers to statistically distinct groups

Strain	Control		6.25 mg L^− 1^			10.0 mg L^− 1^		
	Diameter growth (cm/day)	M.D.	Diameter growth (cm/day)	Inhibition	M.D.	Diameter growth (cm/day)	Inhibition	M.D.
*Gloelophyllum striatum* MCA2	0.69 ± 0.08^a^	2	0.36 ± 0.02^b^	48%	3	0.32 ± 0.01^b^	53%	3
*Datronia caperata* MCA5	0.73 ± 0.08^a^	2	0.24 ± 0.02^b^	67%	1	0.24 ± 0.01^b^	67%	1
*Trametes modesta* MCA6	1.07 ± 0.16^a^	2	0.43 ± 0.05^b^	60%	3	0.36 ± 0.01^b^	66%	3
*Gloelophyllum striatum* MCA7	0.99 ± 0.04^a^	2	0.38 ± 0.02^b^	61%	3	0.38 ± 0.04^b^	61%	3
*Polyporus tenuiculus* MCA9	0.42 ± 0.01^a^	3	0.24 ± 0.02^b^	42%	3	0.22 ± 0.00^b^	48%	3
*Polyporus tenuiculus* MCA11	0.82 ± 0.02^a^	3	0.41 ± 0.01^b^	49%	3	0.32 ± 0.01^c^	60%	3
*Pycnoporus sanguineus* MCA16	1.41 ± 0.05^a^	3	0.45 ± 0.04^b^	68%	3	0.44 ± 0.01^b^	69%	3
Agaricales MCA17	0.85 ± 0.06^a^	3	0.40 ± 0.04^b^	52%	2	0.43 ± 0.01^b^	49%	2
*Polyporus* sp. MCA128	1.56 ± 0.14^a^	3	0.44 ± 0.09^b^	72%	3	0.40 ± 0.02^b^	74%	3
*Hexagonia hirta* MCA131	2.99 ± 0.19^a^	1	1.24 ± 0.02^b^	58%	1	1.16 ± 0.05^b^	61%	1
*Datronia stereoides* MCA167	1.52 ± 0.03^a^	3	0.41 ± 0.02^b^	73%	3	0.38 ± 0.01^b^	75%	3
*Dacryopinax elegans* SXS323	3.63 ± 0.41^a^	3	1.56 ± 0.15^b^	57%	3	1.60 ± 0.21^b^	56%	3
*Pluteus cubensis* SXS320	1.90 ± 0.06^a^	3	1.07 ± 0.01^b^	44%	3	0.73 ± 0.01^b^	62%	3

In 20-day liquid cultures, the biomass production also decreased for the majority of strains when atrazine was present (25 mg L^− 1^), demonstrating the toxic effects of the herbicide even in low concentrations, when compared to agar plates. Abiotic disappearance of herbicide was not detected, and among 13 isolates evaluated, 11 degraded the herbicide in levels varying from 2.9 to 38.7%; it was possible to distinguish three groups of fungi regarding their competence of degrading atrazine (Table 2). Herbicide metabolites appeared in all the culture extracts in which some atrazine dissipation occurred.

**Table 2:** Fungal biomass, laccase activity and herbicide degradation after 20 days in liquid medium with 25 mg L^−1^ of initial atrazine. Superscript lowercase letters (a-c) represent statistically distinct (*p* ≤ 0.05) groups

Strain	Control		Culture in presence of 25 mg L^− 1^ of atrazine			
	Biomass (g L^− 1^)	Lac (U g^− 1^)	Biomass (g L^− 1^)	Inhibition of growth (%)	Lac (U g^− 1^)	Degradation (%)
*Gloelophyllum striatum* MCA2	0.93 ± 0.01	–	0.52 ± 0.06	45	–	0.0^c^
*Datronia caperata* MCA5	0.79 ± 0.06	25.0 ± 8.50	0.64 ± 0.04	19	75.7 ± 5.39	10.5^bc^
*Trametes modesta* MCA6	1.70 ± 0.16	–	0.93 ± 0.02	45	–	14.1^bc^
*Gloelophyllum striatum* MCA7	1.30 ± 0.06	–	1.377 ± 0.08	0	–	37.3^a^
*Polyporus tenuiculus* MCA9	0.97 ± 0.01	–	0.62 ± 0.10	37	–	4.42^c^
*Polyporus tenuiculus* MCA11	1.71 ± 0.47	2.41 ± 1.66	0.85 ± 0.09	51	32.2 ± 11.55	13.9^bc^
*Pycnoporus sanguineus* MCA16	1.47 ± 0.08	9.89 ± 6.70	1.01 ± 0.11	32	15.18 ± 1.11	11.4^bc^
Agaricales MCA17	0.78 ± 0.06	4.03 ± 0.91	0.71 ± 0.04	0	–	38.7^a^
*Polyporus* sp. MCA128	5.07 ± 0.08	–	4.62 ± 0.11	19	–	0.0^c^
*Hexagonia hirta* MCA131	2.11 ± 0.21	–	1.35 ± 0.28	36	–	8.6^c^
*Datronia stereoides* MCA167	5.52 ± 0.03	–	5.04 ± 0.32	18	–	2.9^c^
*Pluteus cubensis* SXS320	1.04 ± 0.16	–	0.94 ± 0.05	10	–	30.0^ab^
*Dacryopinax elegans* SXS323	2.39 ± 0.01	–	1.43 ± 0.12	40	–	3.8^c^
Not inoculated medium (abiotic control	0	0	0	0	0	0.0^c^

The strains *Gloelophyllum striatum* MCA7 and *Agaricales* MCA17 achieved the higher biodegradation levels, 37.3 and 38.7%, and their growth was not inhibited by atrazine in surface cultivation medium. *Polyporus* sp. MCA128, *Datronia stereoides* MCA167, *Pluteus cubensis* SXS320, and *Datronia caperata* MCA5, on the other side, were slightly inhibited by atrazine and had a widely variable degradation performance (Table 2).

*Polyporus tenuiculus* MCA11 showed the strongest laccase induction in the medium containing atrazine, and *Pluteus cubensis* SXS320 exhibited the best degradation rates, while no ligninolytic enzyme had been produced in any condition. Both strains were also among the ones whose growth was less inhibited in PDA (potato dextrose agar) plates (Tables 1 and 2). Therefore, more detailed studies focusing on the atrazine biodegradation and the influence of nitrogen availability on its metabolism were carried out for these two fungal isolates.

Atrazine dissipation by *P. tenuiculus* MCA11 appeared to be only the result of biomass development and cultivation time. This strain removed 39.1% of initial atrazine after 40 days in culture medium with 25 mM of nitrogen, producing 2.24 g L^− 1^ of biomass (data not shown); in a culture containing 2.5 mM of N, the degradation reached 13.9% (with 0.84 g L^− 1^ of biomass) after 20 days of cultivation (Table 2). Atrazine sorption to the mycelium was below 3%.

*Pluteus cubensis* SXS320, by its turn, exhibited a peculiar profile of biotransformation, closely related to nitrogen availability. A more intense degradation of atrazine was achieved by this strain in nitrogen-poor medium, reaching 30% of removal after 20 days of cultivation. Conversely, only 21% were removed after 40 days in a nitrogen-rich medium. Biomass was smaller in nitrogen-deficient cultures (0.943 g L^− 1^, Table 2), while cultures with high nitrogen levels produced 1.6 g L^− 1^ of mycelium on the 40th day of cultivation. Atrazine sorption to the biomass was below 5%.

**Fig. 2:**
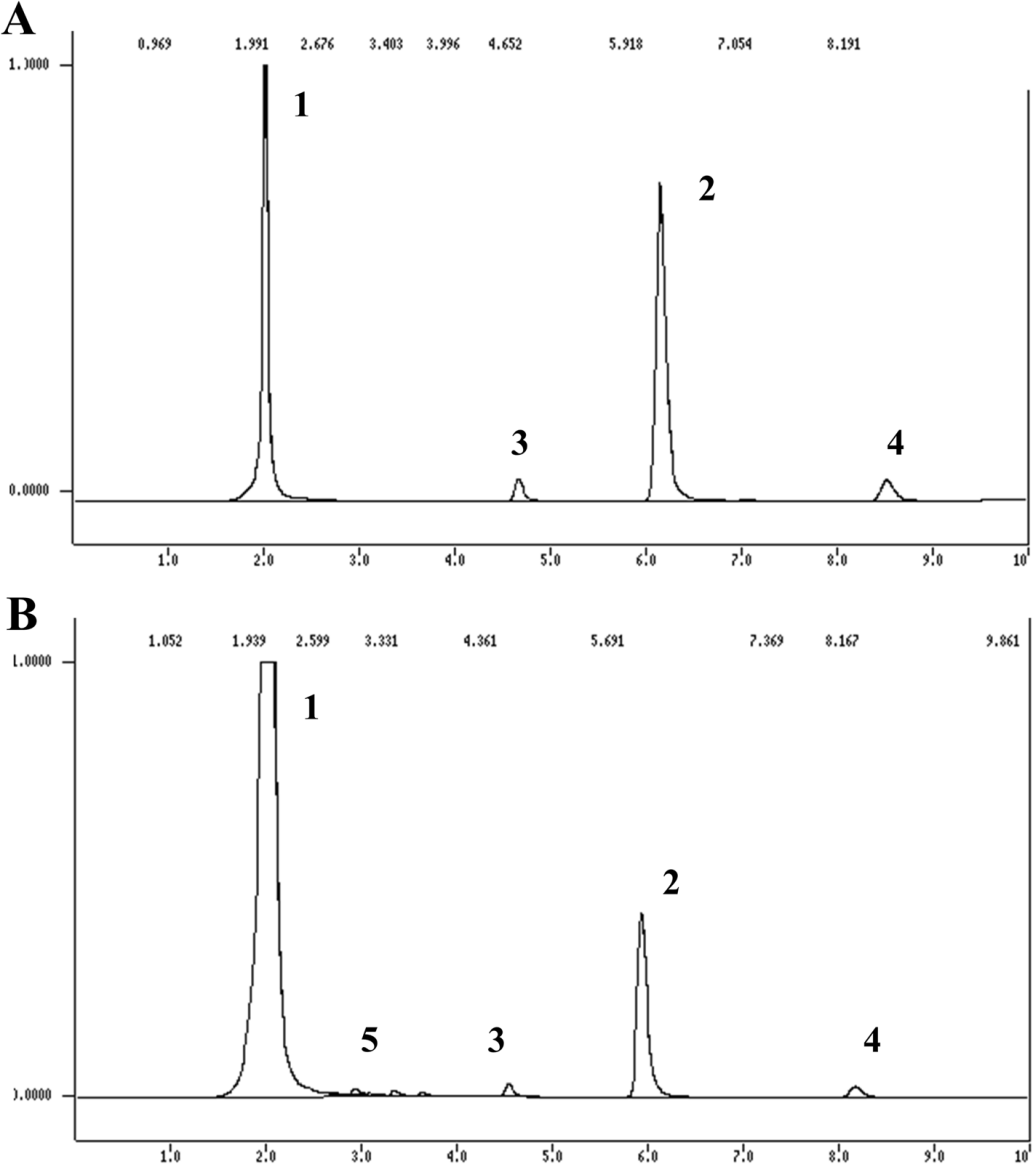
Chromatograms of 40-day culture medium containing 25 mM of N and initial 25 mg L^− 1^ of atrazine. **a** abiotic medium; **b**: *P. cubensis* SXS320 culture extracts. Peak 1: non-identified compound; peak 2: atrazine; peaks 3 and 4: contaminants inherent to commercial formulation employed; peaks 5: metabolites of atrazine

**Fig. 3:**
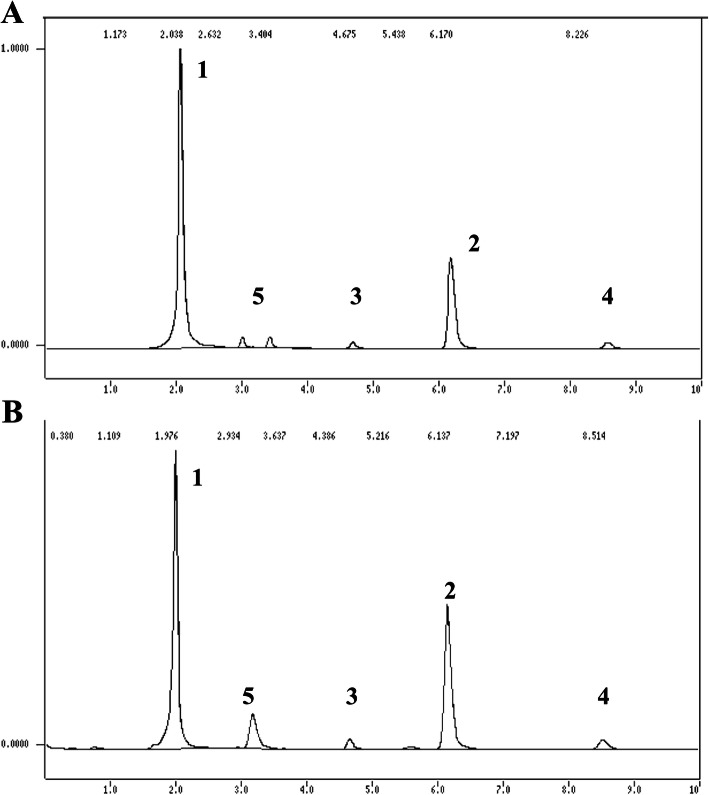
Chromatogram of a 20-day culture of *P. cubensis* SXS320 (**a**) and of *P. tenuiculus* MCA11 (**b**), cultured with 2.5 mM of N. Peak 1: non-identified compound; peak 2: atrazine; peaks 3 and 4: contaminants inherent to commercial formulation employed; peak 5: metabolites of atrazine. All cultures had initially 25 mg L^− 1^ of atrazine

**Fig. 4:**
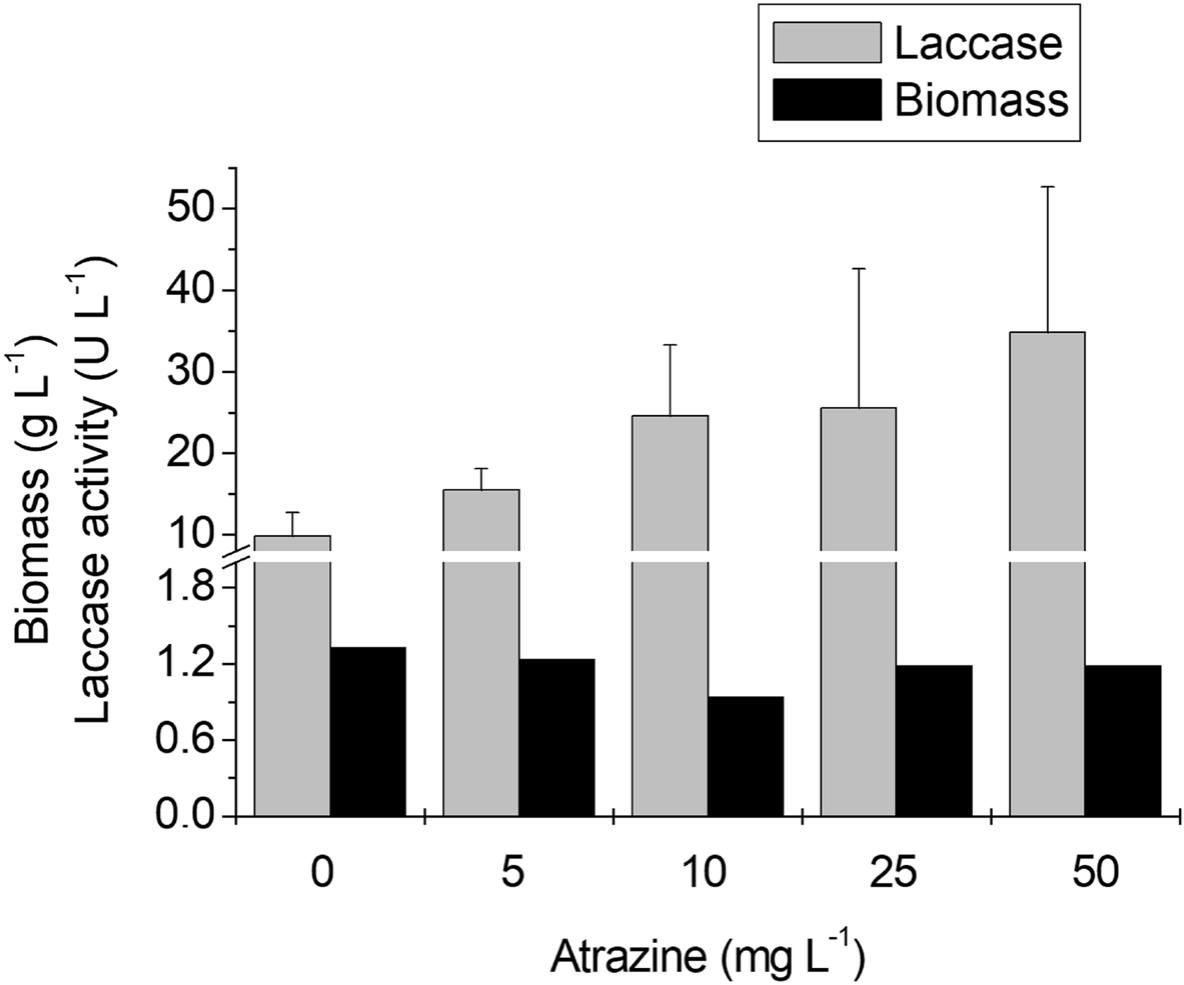
Laccase production by *P. tenuiculus* MCA11; growth supported by 1% of glucose and 25 mM of N, in different atrazine concentrations

Both strains evaluated were able to convert atrazine it into more polar metabolites (Figs. 3 and 4 - peaks 5). The identity of the remaining metabolites, however, was directly affected by the nitrogen availability. In nitrogen rich medium, three metabolites, corresponding to the peaks with retention times of 2.98, 3.6 and 3.40 min, arose in the culture extracts of *P. cubensis* SXS320 within 40 days of cultivation (Fig. 2b). The chromatogram of 40-day culture of *P. tenuiculus* MCA11 had the same profile (not shown). In low-nitrogen medium, however, two different metabolites, whose peaks have retention times of 2.99 and 3.40 min, resulted from the *P. cubensis* SXS320 metabolism of atrazine (Fig. 3a); *P. tenuiculus* MCA11, by its turn, produced only one peak (peak 5, retention time of 3.16 min, Fig. 3b) under the same conditions.

To further explore the relationship among nitrogen availability, atrazine degradation and laccase biosynthesis, we cultivated *P. tenuiculus* MCA11 with atrazine (from 0 to 50 mg L^− 1^), and high N concentration (25 mM). Laccase was slightly induced by increasing atrazine levels, reaching a 4-fold activity level in medium with 50 mg L^− 1^ of the herbicide, compared to the control (Fig. 4). This induction was lower than in the medium with 2.5 mM of N, where laccase levels increased 13-fold in the culture with 25 mg L^− 1^ of atrazine, compared to the herbicide free culture medium (Table 2).

**Fig. 5:**
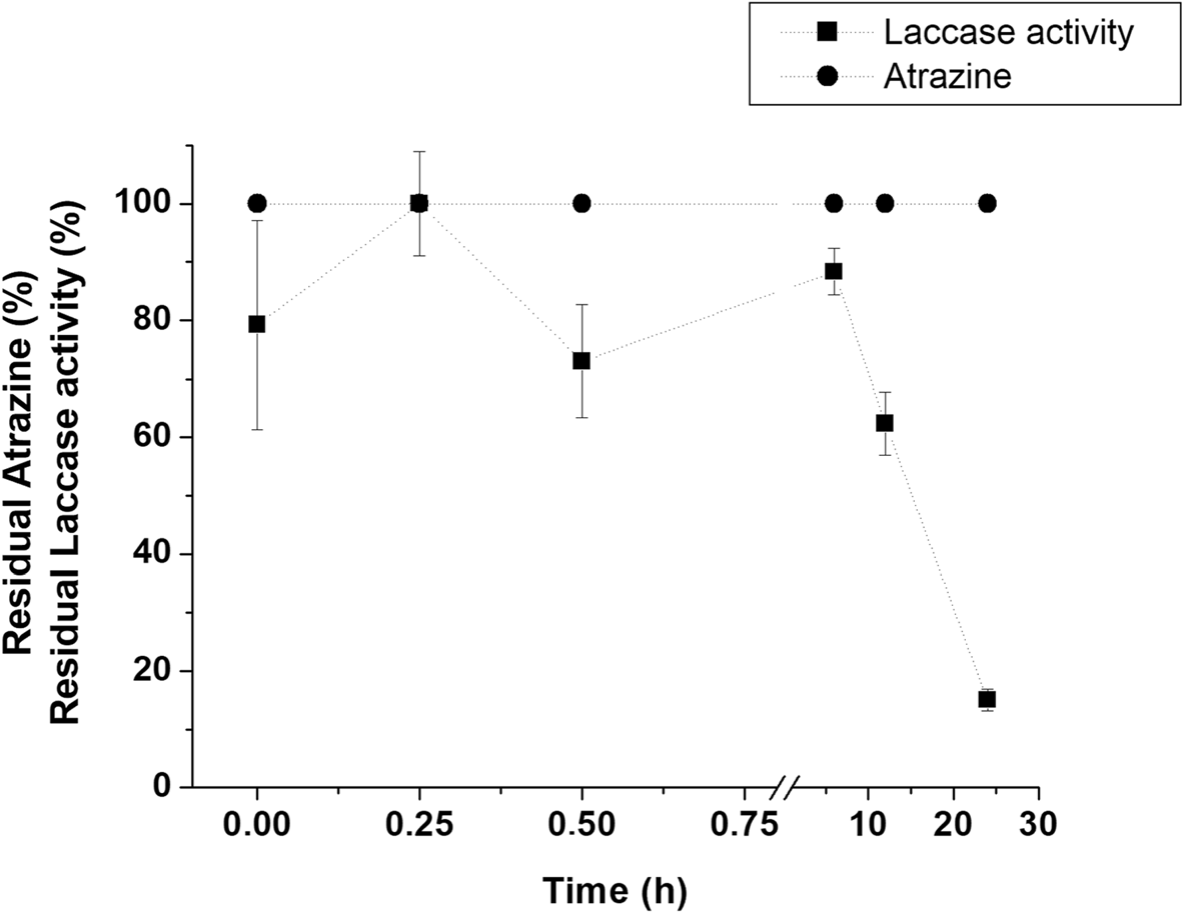
*Polyporus tenuiculus* MCA11 laccase activity and atrazine content, during in vitro incubation

Laccase from *P. tenuiculus* MCA11 was the most strongly induced enzyme during this study; hence, tests to evaluate its potential to degrade the atrazine in vitro were carried out for 24 h. Although laccase had retained 88% of initial activity after 6 h of incubation, no atrazine disappearance occurred (Fig. 5).

## Discussion

Tolerance essays are usually the first step when screening microbial species for bioremediation, once the microorganism needs to overcome the physiological stress caused by the xenobiotic, being able to colonize the contaminated soil or substrate. Previous works reported high inhibition rates when screening fungal strains based on their capacity of growing in agar plates containing organochlorinated pesticides. Growth inhibition ranging from 3.68 to 40.1% were found among 10 white-rot fungi evaluated by Bisht et al. [25], cultured in soil extract agar containing 100 mg L^− 1^ of endosulfan. Dealing with four strains of the basidiomycete genus *Phlebia*, Xiao & Kondo [26] observed growth inhibition from 30 to 60.3% in PDA medium with 50 mg L^− 1^ of lindane. Only *Pleurotus* sp. and *Cymatoderma elegans*, from eight basidiomycetes evaluated by Cupul et al. [10] presented inhibition rates below 75% in plates containing 3.75 g L^− 1^ of atrazine. Given the higher levels of atrazine evaluated here, and the comparable inhibition responses (42 to 73%, in cultures containing 6.25 g L^− 1^ of atrazine), the studied strains can be considered highly tolerant to the herbicide.

Changes in growth rates, pigmentation and spore production are common in fungi growing in toxic environments [27]. After cultivating *Mucor plumbeus* on pentachlorophenol contaminated medium, Carvalho et al. [28] observed an overexpression of proteins associated to increased energy demand, changes on cell wall structure and cytoskeleton, as well as responses to oxidative stress (like cytochrome c oxidase and HSP70 chaperones). The morphological changes observed here, in terms of mycelium density (Table 2) can be the result of similar biochemical disruptions.

Considering atrazine biodegradation potential by fungi, some strains of *Phanerochaete* and *Pleurotus* stand out. *Phanerochaete chrysosporium* removed 48% of initial atrazine from the medium within 4 days [16]. Lopes et al. [4] reported a noticeable performance of *Pleurotus ostreatus* INCQS 40310 degrading 82 and 56% of initial 10 mg L^− 1^ of atrazine and desethylatrazine, respectively, after 15 days in liquid medium; it was the first work demonstrating the biodegradation of atrazine metabolites DIA and DEA by fungi. The proteome analysis indicated that, in this case, extracellular hydrolases and peroxidases were overexpressed in the extracellular environment. Marinho et al. [29] found that atrazine degradation by *Aspergillus niger* in batch reactors containing wastewater and 30 mg L^− 1^ of the herbicide reached 40% after 8 days. The biodegradation rose up to 72% when the mixture was supplemented with 3 g L^− 1^ of glucose, allowing more extensive biomass development.

Once white-rot fungi are expected to grow better in solid substrates and soil, *Anthracophyllum discolor* immobilized in organic supports was capable of degrading 96% of initial atrazine in a soil matrix amended with wheat straw [20]. Considering the first results obtained in this study using liquid medium, strains like *G. striatum* MCA7 and *Agaricales* MCA17, that degraded, respectively, 37.3 and 38.7% of atrazine in liquid culture, have a great potential for extensive atrazine biodegradation in solid matrices, employing biostimulation besides a bioaugmentation strategy. Lignocellulosic crop residues, especially sugarcane bagasse, are abundant bulk agents to support fungal growth in this context.

Previous research found that atrazine degradation by *Phanerochaete chrysosporium* and *Pleurotus ostreatus* INCQS 40310 had a proportional dependence on biomass and associated enzymes [4, 15, 16]. Thus, a nutrient rich culture medium, allowing substantial biomass development, provides best results in terms of biodegradation [22]. However, when ligninolytic system is implicated, it is often subject to catabolic repression in a nitrogen-rich culture medium [21, 30]. Many evidences are in according with this; it was the case of the degradation of aldrin and dieldrin by strains of *Phlebia* [30], and the degradation of atrazine by *Coriolus hirsutus* and *Cerrena maxima* [31]. Tests of ligninolytic activity are often used for selecting microorganisms for bioremediation of aromatics [5]. In fact, laccases, lignin peroxidases and manganese peroxidases can co-metabolyze, in vitro and in vivo, pollutants like PAHs, polychlorinated phenols, polymers, dioxins, and pesticides [19, 32–34].

Even though *P. cubensis* SXS 320 did not produced laccases, lignin peroxidases or manganese peroxidases in any culture condition evaluated here, nitrogen availability modulated the atrazine degradation routes. One hypothesis points to the involvement of some not measured extracellular ligninolytic enzyme, like chloroperoxidase, glyoxal oxidase, tyrosinases and haem-thiolate peroxygenases [5, 35]. Even if the biodegradation depends primarily of cell-bound enzymes, they were also subject of down regulation by nitrogen, once the nitrogen-starving culture medium (that included a poor mycelium development) provided the best results.

Analyzing the behavior of the extracellular laccase produced of *P. tenuiculus* MCA 11 considering the nitrogen and atrazine levels in the culture medium, the results suggest that the biosynthesis of this enzyme is induced by atrazine, in agreement with the previous findings [36, 37]. Besides this, the enzyme is repressed by nitrogen, while herbicide degradation rates were more influenced by mycelium development than laccase levels.

The changes in the profile of the metabolites arising in low or high nitrogen culture medium support the hypothesis of different enzymes acting in each condition. Studying the gene expression of *Phanerochaete chrysosporium*, Kameshawar & Qin [22] found that ligninolytic enzymes were overexpressed in a nitrogen limited medium, while cytochrome P450 enzymes, often crucial for the detoxification of xenobiotics, were mostly overexpressed in nitrogen rich cultures. This means that probably cell-bound constitutive detoxification mechanisms prevail when these strains grow in nutrient rich medium. Once under nitrogen starving, different enzymes, possibly part of the ligninolytic system, are expressed, changing the profile of metabolites [5, 22].

A recent study employing laccase from *Trametes versicolor* reports a poor performance in atrazine removal (less than 5% after 24 h), unless a mediator is used. When 1 mM of HBT (1-hydroxy benzotriazole) was provided, degradation rates improved by 90% [38]. Although ABTS was employed as mediator in the present study, *P. tenuiculus* MCA 11 laccase was unable to oxidize atrazine in vitro. Once ABTS and HBT are the most employed mediators to evaluate atrazine degradation by laccases, taking in account the redox potential of the chemicals involved, these results suggest that this extracellular laccase is not implicated on the atrazine biodegradation, at least on its first steps.

## Conclusion

Previous studies have shown how atrazine biodegradation rates and/or ligninolytic enzymes yields depend on the nitrogen availability in culture medium; but the metabolic change induced only by the availability of this macronutrient, resulting in different biodegradation products for atrazine, was not reported until now. Besides this, the basidiomycete strains evaluated here showed a high potential for bioremediation. The identification of the metabolites resultant from fungal degradation of atrazine, while evaluating strategies to make feasible the further steps towards its complete degradation, are key aspects to ensure the security of future field applications.

## Methods

### Microorganisms

We used basidiomycete strains isolated by Santos et al. [39] and Abrahão et al. [40] from decaying wood from the Atlantic Semi-deciduous forest fragments at “Noroeste Paulista Ecological Station”, Northwestern São Paulo, Brazil. Thirteen strains were chosen based on previous studies screening strains capable of growing quickly and decolorizing Remazol Brilliant Blue as an indicator of ligninolytic activity. Fungi **were** preserved in sterile distilled water [41] in the culture collection of the Laboratory of Biochemistry and Applied Microbiology of “Instituto de Biociências, Letras e Ciências Exatas – IBILCE” from Universidade Estadual Paulista - UNESP.

### Fungal tolerance to atrazine

A commercial formulation of atrazine (Nortox® 500SC, 50% atrazine m/v) was used to test fungal tolerance and biodegradation. The herbicide was added to liquid and warm potato dextrose agar (PDA) medium, to reach the mean and the higher concentrations recommended by the manufacturer for the product when ready to be applied in the soil (6.25 g L^− 1^ and 10.0 g L^− 1^). The medium was homogenized and placed in Petri dishes. Plates were inoculated in the center with 6 mm agar plugs, cut with sterile essay tubes from actively growing cultures in PDA. The cultures were maintained at 28 °C, in the dark, for 15 days. Colony diameters were measured daily, at four equidistant points, and results were expressed as percentage of inhibition related to control (PDA cultures without herbicide). Mycelium density was visually measured and categorized as high, medium, or weak. Results were expressed as mean of three replicates for each treatment.

### Inoculum for liquid cultures

**Fig. 6:**
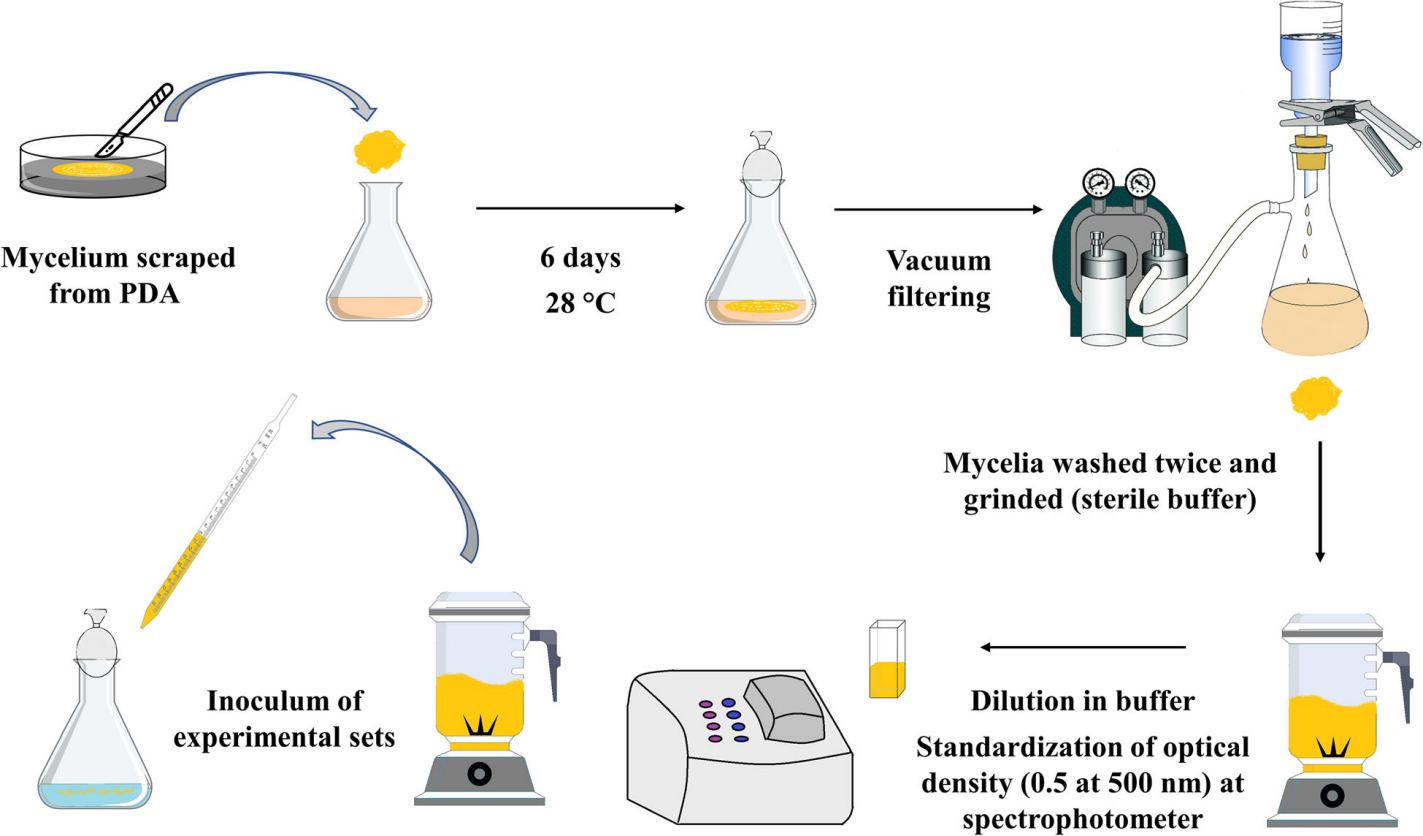
Workflow of the inoculum production and standardization for use in the liquid culture essays

To standardize inoculum size in liquid culture experiments, a cell suspension was produced. Mycelia fragments were scraped from growing cultures in PDA medium and transferred to 250 ml Erlenmeyer flasks containing 50 ml of synthetic culture medium, containing per liter of distilled water: KH_2_PO_4_, 0.2 g; MgSO_4_ ∙ 7 H_2_0, 0.05 g; CaC1_2_, 0.01 g; mineral solution, 1 ml; and vitamin solution, 0.5 ml (to details of mineral and vitamin solution, see Kirk et al. [23]). The medium was supplemented with 1% of glucose and NH_4_NO_3_ to supply 25 mM of nitrogen. After 6 days at 28 °C, cultures were drained; mycelium was washed twice with sterile Knapp buffer [42] and ground on a stainless steel sterile mixer with the buffer. The resultant cell suspension was diluted in buffer to reach an optical density of 0.5 at 550 nm (DU Spectrophotometer, Beckmann), and then used immediately to inoculate test cultures (Fig. 6).

### Atrazine degradation and enzyme production

An atrazine stock solution was prepared by homogenizing the commercial formulation in methanol, followed by two sonication pulses of 15 min and centrifugation at 10,000 g for 10 min. Supernatant was filtered through a Millipore membrane of 0.2 μm pore size. Aliquots were dispensed into sterile 125 ml Erlenmeyer flasks to reach final concentrations of 0 to 50 mg L^− 1^. After 24 h, checking for the complete methanol evaporation, 25 ml of sterile culture medium described above were added. The medium was supplemented with 1% glucose and NH_4_NO_3_ (to reach 2.5 mM of N for a nitrogen-deficient medium). Each flask was inoculated with 1 ml of the cell suspension. Cultures were vacuum filtered after incubation for 20 days, under static conditions at 28 °C. The supernatant, referred as crude extracts, were kept in an ice bath for enzyme assays, and aliquots were frozen. Mycelia were dried at 70 °C to evaluate biomass and fragments were reserved to measure fungal atrazine sorption. Sterile controls were included. Results are express as mean of three replicates under each condition [43].

Aiming to evaluate the influence of nitrogen upon atrazine degradation, the strains *Polyporus tenuiculus* MCA11 and *Pluteus cubensis* SXS320 were cultivated as described above, supplementing the culture medium with 25 mM of N (nitrogen sufficiency condition). Enzymatic essays were done after 8, 24, 16, 32 and 40 days of cultivation. Biomass and atrazine degradation were evaluated at the end of cultivation time.

### Atrazine biotransformation analysis by high performance liquid chromatography (HPLC)

Frozen samples from cultures were melted, centrifuged at 10,000 g for 10 min and filtered in a 0.22 μm pore size filter. Then, 20 μL of each sample were injected into a Jasco HPLC, by using a C18 Perkin Elmer column contained in an oven at 40 °C. Pesticides and metabolites were eluted by using a mobile phase of acetonitrile:water (55/45 v/v) with a flow rate of 1 ml min^− 1^, and detected by UV absorbance at 220 nm. Analytical standard was purchased from Sigma (atrazine, 98% of purity). Quality control of the HPLC data was based on the standard curve for concentrations between 1 up to 50 mg L^− 1^ (R^2^ = 0.992). The recovery index (99%) was based on the detected atrazine amounts after diluting known amounts of the herbicide in the culture medium employed in the essays. To evaluate sorption of atrazine to fungal mycelium, the biomass (approximately 150 mg) was ground in a mortar and extracted twice with 500 μL of n-hexane. Extracts were combined and air dried. The residue was re-suspended into 1 ml of mobile phase, filtered through 0.22 μm pore filter, and analyzed in chromatograph (adapted from Vroumsia et al. [44]).

### Enzyme assays

Laccase activity was measured throughout the reaction using 0.9 ml of sodium acetate buffer (10 mM, pH 3.5) with 0.03% of ABTS - 2,2′-azino-bis (3-ethylbenzothiazoline-6-sulfonic acid) diammonium salt (Fluka, Switzerland), and 0.1 ml of diluted enzyme crude extract [38]. After incubation for 1 min at 40 °C, the absorbance was read at 420 nm [21]. For the reaction blanks, water replaced enzyme extract. Reaction mixtures of controls lacked ABTS. One unit of activity represents the amount of enzyme that oxidizes 1 μmol of ABTS per minute, considering ε_420_ = of 3,6.10^4^ M^− 1^ cm^− 1^ for oxidized ABTS. The activity was expressed as U g^− 1^ of dry biomass.

Analysis for manganese-dependent peroxidases were carried out by adding 0.1 ml of crude enzyme extract into a mixture containing 0.8 ml of sodium lactate buffer (50 mM, pH 4.5), 0.1 ml of MnSO_4_ solution (400 mM) and 4 μL of a 10 mM H_2_O_2_ solution. After 10 min incubated at 40 °C, readings were made at 240 nm [45]. In the blanks, enzyme was replaced by distilled water, while controls lacked MnSO_4_. One unit of enzyme activity represented the enzyme necessary to create 1 μmol of complexes lactate-Mn^3+^ per minute, considering ε_240_ = 8,1.10^3^ M^− 1^ cm^− 1^ [46].

Reaction for lignin peroxidases was based on the oxidization of veratryl alcohol into veratraldehyde. Enzymatic extract was added to a mixture containing 0.8 ml of sodium tartarate buffer (pH 3.0, 50 mM), 0.1 ml of a 40 mM veratryl alcohol (Fluka, Switzerland), and 4 μL of 100 mM H_2_O_2_ [47]. After 10 min at 40 °C, absorbance was read at 310 nm. In the blanks, distilled water replaced the enzyme; in controls, veratryl alcohol was replaced by water. One unit of enzyme activity represents the amount of enzyme that oxidizes 1 μmol of veratraldehyde per minute, using ε_310nm_ = 9.10^3^ M^− 1^ cm^− 1^.

### Effects of laccase upon atrazine in vitro

A reaction mixture containing 0.8 ml of a 25 mg L^− 1^ atrazine solution, 0.1 ml of a 500 mM sodium acetate buffer (pH 2.5) with 0.03% of ABTS and 0.1 ml of *P. tenuiculus* MCA11 enzyme crude extract was incubated at 40 °C for 24 h (optimal pH and temperature for laccase activity were determined in previous essays). Periodically, aliquots were taken and immediately frozen in liquid nitrogen. For the analysis of atrazine content, melted samples were filtered through a 0.22 μm membrane and evaluated by HPLC. Reactions employing pH 7.0 (McIlvaine buffer) or omitting ABTS, enzyme or atrazine were included as controls [19].

### Data analysis

One-way ANOVA (analysis of variance), followed by Tukey *post-hoc* tests, was used to test the influence of atrazine upon fungal growth and enzyme production, as well as atrazine biodegradation rates. Normality and homoscedasticity were tested using Shapiro-Wilk and Levene’s test, respectively. Analyses were conducted in the software R Studio version 3.5.1 (R Core Team [48]).

## Data Availability

The datasets analyzed during the current study are available upon request to the corresponding author.

## Abbreviations

ABTS: 2,2′-Azino-bis (3-ethylbenzothiazoline-6-sulfonic acid)
DEA: Deethylatrazine
DEA-DIA: Deethyldeisopropylatrazine
DIA: Deisopropylatrazine
HA: Hydroxyatrazine
HBT: 1-Hydroxy Benzotriazole
HPLC: High Performance Liquid Chromatography
Lac: Laccase
LiP: Lignin Peroxidase
MnP: Manganese Peroxidase
PAHs: Polycyclic Aromatic Hydrocarbons
PDA: Potato Dextrose Agar
